# A Prospective Randomized Study Comparing Functional Outcome in Distal Fibula Fractures between Conventional AO Semitubular Plating and Minimal Invasive Intramedullary “Photodynamic Bone Stabilisation”

**DOI:** 10.3390/jcm11237178

**Published:** 2022-12-02

**Authors:** Michael Zyskowski, Markus Wurm, Frederik Greve, Philipp Zehnder, Patrick Pflüger, Michael Müller, Peter Biberthaler, Chlodwig Kirchhoff

**Affiliations:** Department of Trauma Surgery, Klinikum Rechts der Isar, Technical University of Munich, Ismaninger Str. 22, 81675 Munich, Germany

**Keywords:** ankle fracture, elderly, fibular, fragility fracture, intramedullary stabilsation, IlluminOss^®^, osteosynthesis

## Abstract

(1) Background: As age in western populations is rising, so too are fractures, e.g., of the distal fibula. The aim of this study was to find out whether a novel, minimally invasive intramedullary osteosynthesis technique for the treatment of distal fibula fractures in elderly patients results in not only a reduction of postoperative complications, but also a shorter hospitalization time, an improved clinical outcome, and preserved autonomy in geriatric trauma patients. (2) Methods: In this prospective study, the results following surgical treatment for distal fibula fractures in geriatric patients after using DePuy Synthes^®^ one-third semitubular plate (Group I) or a minimally invasive intramedullary photodynamic Bone StabilizationSystem (IlluminOss^®^) (Group II) were compared at 6 weeks, 12 weeks, 6 months, and 1 year after initial treatment. (3) Results: Significant improvement regarding clinical outcome was shown in Group II 6 and 12 weeks after surgery. (4) Conclusions: Our study results demonstrate that the use of this new intramedullary stabilization system in combination with an immediate postoperative weight bearing seems to be a safe and stable treatment option for ankle fractures in geriatric patients, especially in the early stages of recovery.

## 1. Introduction

Ankle fractures (FX), common injuries constituting about 9% of all FX [1] of the human skeleton, are considered the third most common FX in geriatric patients [2,3]. In recent years, an increasing number of elderly patients have suffered from ankle FX [4,5]. The majority of these elderly patients already suffered from numerous comorbidities at the time of the accident [6]. The focus of fracture treatment in the elderly population is to achieve as much freedom as possible, in combination with maintaining quality of life comparable to the pre-accidental level. Nevertheless, operative treatment is associated with typical complications, such as mal- or nonunion, and especially skin problems ranging from delayed wound healing to severe skin defects [7,8,9,10,11,12].

Open reduction and internal fixation (ORIF) has become the standard of care for displaced ankle FX in adults [13,14]. Several techniques for internal ankle fixation are commonly used, ranging from simple lag screw fixation to plate osteosynthesis with non-locking to locking screw systems up to biodegradable systems [15,16,17,18,19,20]. In this context, several studies in the common literature focus on intramedullary (IM) fixation of the distal fibula FX, fracture stabilization, and the appearance of soft tissue-related complications in the older population [21,22,23,24,25,26,27].

In addition to fracture healing and the complications related to the surgery itself, postoperative therapy is a prognostic factor for the patient’s satisfaction, functional outcome, and return to normal daily activities [28,29,30,31]. For older patients suffering from an ankle fracture, returning to their normal daily life is important in terms of their quality of life and freedom [32,33,34]. In older patients with preexisting comorbidities, surgical treatment must not only respect the soft tissue conditions, but should also have the goal of a shortened pre- and post-operative therapy time window, shortened hospital stay, faster time of recovery, and achievement of full resilience as quickly as possible [6,35]. Therefore, the main goal of any post-operative therapy is to reduce time of recovery to a minimum and to achieve full physical capacity as early as possible. In the past, a few clinical trials have shown that early weight bearing and functional treatment avoiding a plaster cast may shorten the immobilization period, but it also may provoke a loss of reduction, depending on morphology and initial stability of the FX, as well as patients’ age and comorbidities [28,32,36,37,38,39]. A few clinical trials have demonstrated that IM ankle FX fixation might be a good method to preserve the soft tissue, but it could lead to fibula shortening and loosening of the implant [21,22,26,27,40,41].

Thus far, surgical treatment of FX with IM nailing has already shown promising results in the treatment of various complex fractures of the femur [42], tibial shaft [43], and clavicle [44]; hence, IM nailing has become a standard implant. To the best of our knowledge, there still exists no prospective randomized trial assessing the treatment of distal fibula FX using these modern implants.

In this context, surgical treatment of ankle fractures using IM fixation systems were controversially discussed because of their biomechanical performance and implant costs [45,46].

The aim of this study was to analyze whether open reduction internal fixation (ORIF) of distal fibula FX, using a standard semitubular plate or closed reduction and IM fixation using a new minimally invasive intramedullary Photodynamic Bone Stabilization System (IlluminOss^®^), allows for a better outcome for immediate postoperative weight bearing and the reduction of complications in older patients with a wide range of comorbidities.

## 2. Materials and Methods

### 2.1. Patients

**Figure 1:**
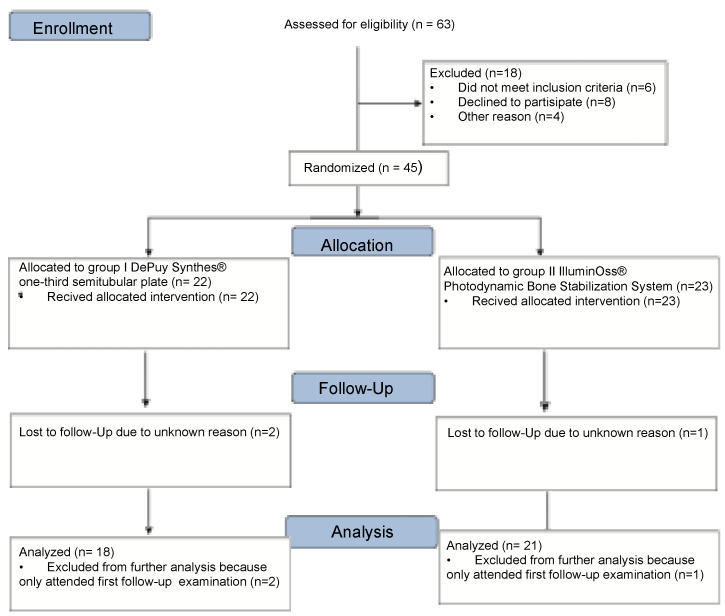
CONSORT (Consolidated Standards of Reporting Trials) flow diagram.

Institutional Review Board approval was obtained prior to this study (IRB approval No: 103/15, Ethical Committee of Technical University Munich, Registration in the German Clinical Trails Register trail number DRKS00025496). This was a prospective randomized single center study with two parallel groups. The study was conducted under consideration of the CONSORT statement (Figure 1). All patients over 65 years suffering from a distal fibula FX according to AO classification (AO 44 B1.1, B1.2, B1.3) with consecutive indication for surgery and a Charlson Comorbidity Index (CCI) greater than one who presented at our academic level-one trauma center between 06/2015 and 06/2018 were prospectively enrolled [47,48]. The CCI predicts the 10-year survival in patients with multiple comorbidities and takes into consideration 19 comorbid diseases, including cardiovascular disorders, diabetes mellitus, liver and lung diseases, cerebrovascular incident and transient ischemic attacks, dementia, COPD, and connective tissue diseases [47]. Written informed consent of the patients was obtained. A randomization plan (Randlist^®^, DatInf GmbH, Tübingen, Germany) was used to divide patients regarding treatment using either the DePuy Synthes^®^ one-third semitubular plate (DePuy Synthes^®^, Umkirch, Germany) (Group I) or the IlluminOss^®^ Photodynamic Bone Stabilization System (llluminOss Medical, Inc., East Providence, RI, USA) (Group II).

Exclusion criteria were mental disorders, patients under comprehensive legal support, and pathological or open FX [40].

### 2.2. Surgical Technique and Postoperative Therapy

All patients were operated on by expert trauma surgeons, experienced in lower extremity surgery. After assessment by the anesthetists, general anesthesia was performed in all cases. A single prophylactic dose of 1.5 mg cephalosporin was administered preoperatively. The patients were placed in a supine position on a radiolucent table with a pillow under the ipsilateral gluteal region and the injured ankle.

In group I, all surgeries were performed under tourniquet control (250 mmHg) using a standard lateral approach to the distal fibula according to AO recommendations. ORIF was performed according to AO guidelines using a 3.5 mm lag screw and semi-tubular neutralization plate, as well as 3.5 mm cortical screws [41]. The syndesmotic stability was verified with the lateral hook test [49].

Group II required preoperative preparations. The IlluminOss^®^ Photodynamic Bone Stabilization System needs hardening by visible light (436 nm). The length of the light fiber cable is predetermined to 183 cm. Therefore, the light box is placed outside of the sterile surgical field on the fractured side, but as close as under sterile surgery conditions possible to assure the connection between the light cable and light box.

**Figure 2:**
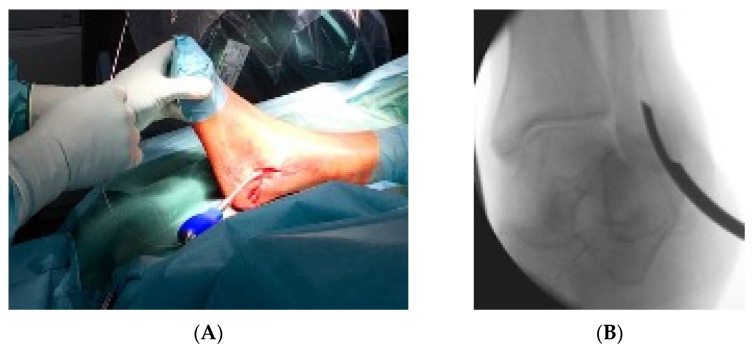
(**A**) demonstrates the insertion of the 4 mm cannulated awl with the corresponding intraoperative fluoroscopic control (**B**).

**Figure 3:**
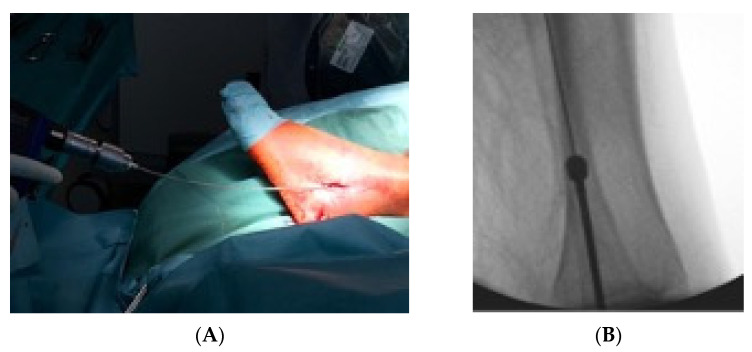
In (**A**) the 1 mm stepwise reaming with a minimum diameter of 4.5 mm is shown with the corresponding intraoperative fluoroscopic image of the reaming procedure (**B**).

**Figure 4:**
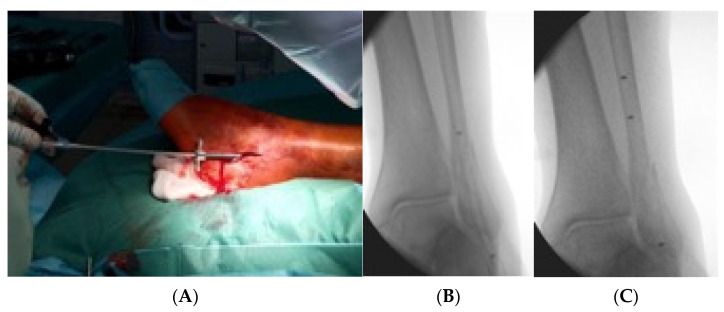
This figure demonstrates the insertion of the implant into the fibula via the already placed sheath (**A**), the fluoroscopic control of the insertion (**B**) and confirmation of the final position via fluoroscopic control and radiopaque markers on the implant (**C**).

**Figure 5:**
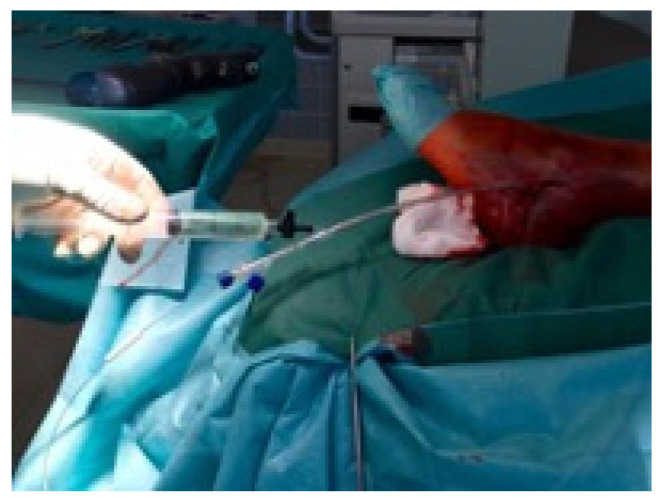
This figure shows the infusion of the balloon with the monomer.

**Figure 6:**
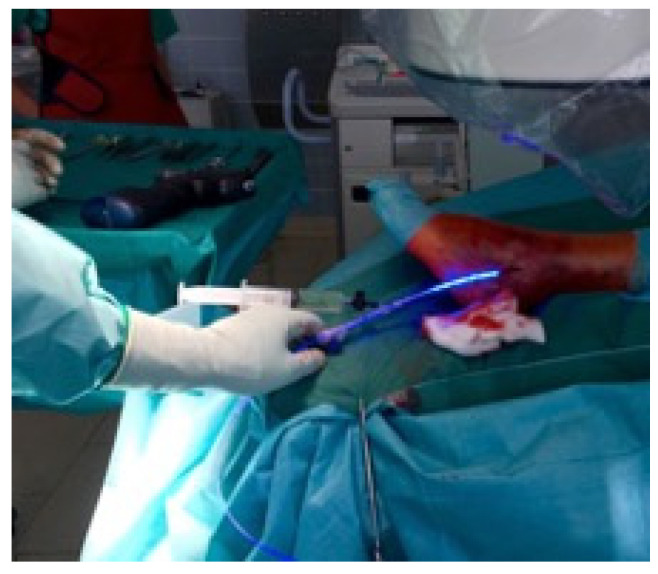
Here, the curing of the monomer by the visible light of 436 nm is shown.

Primarily the closed reduction of the fracture was performed using image intensifier control in two planes. Then, the tip of the fibula was incised using a straight awl under fluoroscopy control. After the correct position was achieved, the entry portal was enlarged. Then, the straight awl was removed, followed by the insertion of a 4 mm cannulated awl (Figure 2A,B) allowing for the insertion of a ball tip guidewire bridging the FX into the medullary canal. Consecutively, the cannulated awl was removed. The ball tip guidewire assured that the now inserted cannulated burrs for reaming the medullary canal stayed in the designated position to be able to clear the fracture site. Reaming was performed in 0.5 mm steps to clean the channel with a minimum diameter of 6 mm up to a maximum diameter of 8.0 mm (Figure 3A,B). To avoid additional fractures of the fibula shaft, additional reamers with a diameter more than 8.00 mm should not be inserted. Furthermore, the surgeon should perform a fluoroscopy while reaming and have experience in intramedullary osteosynthesis. Then, the cannulated burr was removed while the guidewire remained in situ. Consecutively, a dilator and sheath were placed in the fibula under fluoroscopy control. When reaching the correct position, the guidewire and the dilator were removed. During the preceding surgical steps, the OR nurse assembled the implant by removing air from the balloon and transferring the monomer into a syringe. The prepared implant was handed to the surgeon and, finally, the monomer was inserted into the fibula via the already placed sheath (Figure 4A–C). When the right position was confirmed, the sheath was slowly separated and removed. Then the balloon was infused with the monomer (Figure 5). The last two steps were assured under fluoroscopy guidance. When the light cable was handed to the surgeon, the timer was put onto the front of the light box. The specific light emission time was adapted to the length of the chosen implant. The visible light corroborates the monomer (Figure 6); this process should not be disrupted or stopped. After the input hardening time, the light stopped automatically. Then, the catheter connected to the balloon was removed using a slap hammer. As the catheter was removed, the syndesmotic stability was test with the dorsiflexion-external rotation stress test under fluoroscopic control [50]. The last surgical steps include a final fluoroscopic control in two planes, wound closure, and dressing. Postoperatively, physical therapy was initiated. Preoperative ASA Physical Status Classification System was collected from the anesthesia documentation [51].

Group I patients were treated following the rehabilitation protocols (Table 1) of our trauma department, which allowed partial weight bearing restricted to 20 kg for 6 weeks, using crouches or a medical walking boot, and pain-adapted motion out of the walking boot without limitations, according to the recommendations of the German Society for Orthopedics and Trauma (DGOU) [44]. After the initial 6 weeks, these patients were allowed to increase the load of weight bearing with the goal to achieve full weight bearing within 10 weeks after surgery.

In Groupe II, full weight bearing was allowed right after surgery using a medical walking boot without the use of crouches and pain-adapted motion out of the walking boot without limitations. After 3 weeks, the patients were allowed to train the medical walking boot away, with the possibility to switch from the walking boot to an ankle brace.

### 2.3. Follow-Up Evaluation

The first follow-up exam was set up 6 weeks postoperatively. Additional follow-ups were terminated after 3, 6, and 12 months. The follow-up examinations were performed by independent investigators not involved in patients’ initial surgical treatment (PZ, FG, MW) at the outpatient clinic of our level-one university trauma center.

For assessment of pain, the visual analogue scale (VAS) [52], ranging from 0 “no pain” to 10 “worst imaginable pain”, was used. ROM and ligament stability were registered during standardized clinical follow-up examination. Moreover, sensomotoric disorders and postoperative complications were recorded. Minor complications were defined as those possibly treatable conservatively (e.g., superficial wound infections, delayed union etc.), whereas major complications needed operative revision (e.g., secondary loss of reduction, non-union, severe wound infections, etc.).

For the assessment of lower extremity and ankle function, the Olerud and Molander ankle score (OMAS) [53,54] and the Karlsson and Peterson Scoring System for Ankle function (KPSS) [55] were comprised and stated the primary outcome markers.

*X*-rays were taken postoperatively, as well as at the follow-up examinations, and evaluated with special respect to signs of bony healing and secondary loss of reduction.

### 2.4. Statistics and Sample Size Calculation

Statistical analyses were performed using the statistical software SigmaStat (version 3.5; Systat Software, San Jose, CA, USA). The scores at certain time points were compared with an independent *t* test after a normality check was passed and equal variances were detected. Normal distributed data with unequal variances would have been compared using the Welch’s *t* test. Arbitrarily, data were tested with the Mann–Whitney U test. The significance level was set at *p* = 0.05.

Power analysis was performed prior to this study using G*Power for Mac. The Wilcoxon signed-rank test was used to compare the means of pre- and postoperative values. The significance threshold was set at a *p* value of <0.05.

We derived these figures from preliminary studies with OMAS in ankle fractures [56]. At a significance level of 0.05 and using the Welch’s *t* test for independent samples, 20 persons per experimental group are needed to achieve a power of 80%.

## 3. Results

### 3.1. Epidemiological Data

At first, 45 patients were enrolled in this study. The mean age was 77 years (range 65–93 years). Of the ankles fractured, 24 were right (53%) and 21 left (47%). Three patients were lost to follow-up for unknown reasons, whereas 3 patients were only able to attend the first follow-up examination due to further health-related causes. The remaining 39 of 45 patients (86%) available for all follow-up examinations presented with a mean age of 77 years (range 65–92 years) and a CCI of 2 (range 1–3 points) at the time of injury, with no statistical differences between both groups. Eighteen patients were assigned to group I (46%) and 21 patients (54%) to group II.

The most common injury type was ankle sprain resulting from supination and external rotation trauma according to the Lauge Hansen classification [57,58]. All accidents happened during spare time and were of low velocity character (100%).

Regarding gender distribution, 16 male (41%) compared to 23 female patients (59%) were included. The interval between trauma and surgery accounted for an average of 8 days (2–20 days). Group I had an interval time of 9 days (5–20 days) and Group II an interval time of 4 days (2–9 days), which reached statistical significance when compared (*p* = 0.01, see Table 2). Correspondingly, the mean length of stay in hospital accounted for an average of 6.2 days (range 2–21 days): for Group I, 9 days (range 4–21 days) and for Group II, 5.3 days (range 2–8 days), which showed a significant difference (*p* = 0.05, Table 2).

Regarding the side of the fracture, 20 right (51.3%) compared to 19 left (48.7%) ankles were fractured. In Group I, 10 right (55.6%) and 8 left (44.4%) ankles were treated versus treatment of 10 right (47.6%) and 11 left (52.4%) ankles in Group II. Regarding the fracture morphology, no difference in distribution considering the AO classification (AO 44 B1.1, B1.2, B1.3) in the treated patients could be shown for both groups. In Group I, 15 patients (83.3%) showed an AO 44 B1.1 type fracture, 2 patients (11.1%) an AO 44 B1.2, and 1 patient (5.6%) an AO 44 B1.3. A similar fracture morphology distribution was detected in Group II, were 17 patients (81.0%) with an AO 44 B1.1, 2 with an AO 44.B1.2 (9.5%), and 2 with an AO 44 B1.3 (9.5%) were treated. The body mass index (BMI) for both groups was similar, with a BMI of 26 in Group I and a BMI of 24 in Group II (see Table 2). The ASA-Score showed no significant differences between the both groups. In addition, regarding the surgical time, in general anesthesia, no significant differences could be shown, with a slight advantage for Group II (see Table 2).

### 3.2. Clinical Outcome

Only Group I patients (*n* = 4, 22%) presented with minor complications, such as swelling and redness of the wound in two cases (11%) and one deep vein thrombosis (5.5%). In one patient, bronchial pneumonia resulted from a prolonged hospital stay due to difficult postoperative mobilization (5.5%) treated with intravenous antibiotics for one week. Group I showed a significantly higher incidence of minor complications compared to Group II, which did not have any minor complications (*p* = 0.01, see Table 3).

In both study groups, one major complication was observed. In detail, one Group I patient (5.5%) suffered from a deep wound infection resulting in material loosening followed by operative debridement, material removal, and intravenous antibiotics for ten days. One Group II patient (4.7%) showed a loss of reduction such that the operative procedure was changed to the semitubular plate treatment (*p* = 0.47, see Table 3).

During the first clinical follow-up examination, all patients were asked if they had been able to follow the rehabilitation protocol. All patients, regardless of group assignment, stated that they followed the recommended rehabilitation protocol.

The Olerud and Molander ankle score (OMAS) showed in the first two clinical follow-ups after 6 and 12 weeks significantly better results in Group II compared to Group I (*p* = 0.01, see Table 4). Similar results for the first two clinical follow-ups were recognized in Group II for the Karlsson and Peterson Scoring System for Ankle function (KPSS), confirming the good OMAS results (*p* = 0.01 and 0.02, see Table 4). In the later follow-up exams after 6 and 12 months, no further statistical difference between both groups was found (OMAS *p* = 0.06 and 0.07, KPSS *p* = 0.06 and 0.06, see Table 4). Regarding the VAS, only at the first clinical follow up was a statistical difference detected (*p* = 0.01, 0.09, 0.24 and 0.15, see Table 4). Regarding range of motion (ROM), statistically high significant differences could be identified in our clinical follow-up exams between both groups for extension (dorsiflexion) and flexion (*p* = 0.01, see Table 5).

### 3.3. Radiological Follow Up

**Figure 7:**
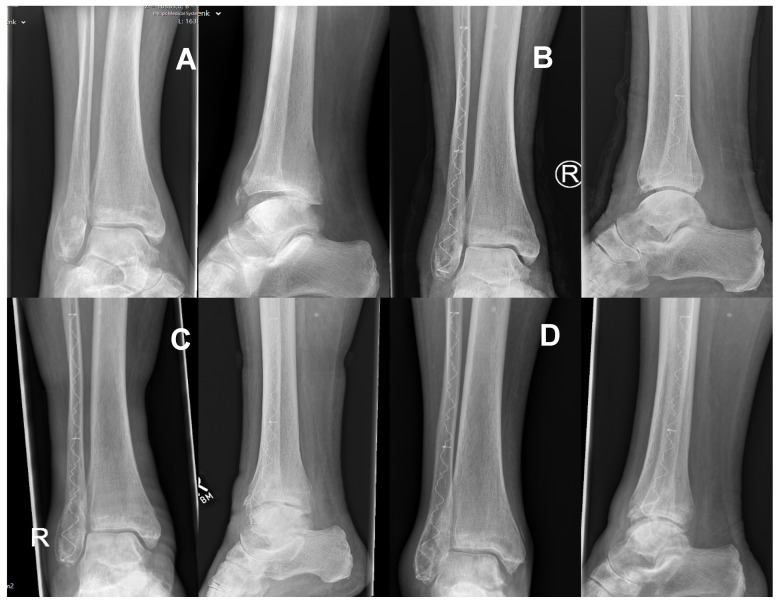
Radiograph of a 77-year-old female patient with an AO type 44 B1.1 ((**A**) a preoperative) fracture treated with the IlluminOss^®^ Photodynamic Bone Stabilization System. Postoperative results after 6 (**B**) and 12 weeks (**C**) as well as after 14 months (**D**) showed excellent fracture healing.

**Figure 8:**
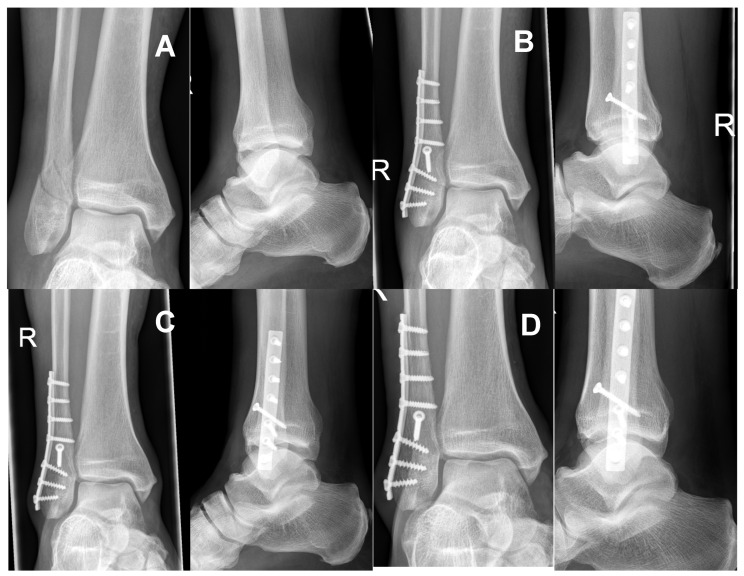
(**A**) shows the preoperative radiograph of a 72-year-old male patient with an AO type 44B1.1 fracture. The patient was treated with the DePuy Synthes^®^ one-third semi tubular plate system. Figure (**B**) presents the postoperative results after 6 and Figure (**C**) after 12 weeks. In addition, the 14 months postoperative control (**D**) shows good fracture healing without any secondary complications.

At the final follow-up (12 months postop), 39 patients—18 treated with the DePuy Synthes^®^ semitubular plate and 21 patients using the IlluminOss^®^ Photodynamic Bone Stabilization System—demonstrated complete osseous healing on radiographs without any signs of complications (see Figure 7 and Figure 8). No case of non-union was identified. The one Group I patient who suffered from a deep wound infection and loosening of the implant needed a conversion to a conservative cast treatment. His fracture healed without non-union 15 months after the initial surgery. For the single Group II patient who showed a loss of reduction, fracture healing without non-union was achieved within 14 months after conversion to a semitubular plate fixation treatment.

## 4. Discussion

To the best of our knowledge, this is the first randomized controlled study to compare semitubular plates with the presented new intramedullary stabilization system in distal fibula fracture treatment with an immediate postoperative weight bearing regime in the intramedullary stabilization patient group. With increasing age, the probability of fracture occurrence rises, and also the number of ankle fractures (9% of all human skeleton fractures [1]) increases; thus, ankle fractures are considered the third most common FX in geriatric patients [2,3]. Usually, many of these elderly patients with ankle FX already suffer from numerous comorbidities at the time of the accident [6]. In this context, treatment of ankle fractures in the elderly population should result in achieving as much freedom as possible, in combination with quality of life comparable to before the accident. Nevertheless, operative treatment is usually associated with typical complications, such as mal- or nonunion, and especially skin problems ranging from delayed wound healing to severe skin defects [7,8,9,10,11,12]. Commonly known open reduction and internal fixation (ORIF) using various techniques is the standard of care for displaced ankle FX in adults [15,16,17]. Generally speaking, a recent advance in the treatment of fractures of different bones was the introduction of intramedullary nails [43,44]. Thus far, the use of intramedullary nailing systems in distal fibula fractures has been investigated under the aspects of FX stabilization, soft tissue management, and complication rates [25,26,27]. The majority of these studies were of retrospective character. The presented prospective randomized study focused on the comparison of treating ankle fractures of patients at an age over 65 years either using a rather established semitubular plate AO-system or an intramedullary Photodynamic Bone Stabilization System (IlluminOss^®^). Our results show that the IlluminOss^®^ system allows for a progressive postoperative rehabilitation protocol performing full weight bearing immediately after surgery, leading to reliable results along with a good functional outcome.

In the general literature, complication rates of up to 25% have been reported for ankle fractures, especially in geriatric patients [10,13]. These complications include wound healing problems, redness, hyperthermia, superficial and deep infections, deep leg vein thrombosis, and loosening of the material resulting in a loss of reposition. Previous studies in elderly patients with preexisting comorbidities demonstrated that intramedullary nailing systems in distal fibula FX have a significantly lower incidence of soft tissue complications and lead to adequate FX stability compared to conventional plate osteosynthesis [26,28,29,30]. White et al. described in their prospective randomized study on 100 patients (*n* = 50 ORIF, *n* = 50 IM nailing) no superficial wound infections in the IM group compared to eight (16%) superficial wound infections in the ORIF group [22]. Similar results for wound infections following IM nailing were described by Cofiman et al. [27]. In their study, only 1 deep infection occurred in the 34 patients available for follow-up after IM nailing [27]. Both studies consider small incisions as beneficial for soft tissue healing [22,27]. In the actual study, the rate of postoperative complications was lower in the Photodynamic Bone Stabilization System group (Group II). Similar to White et al. and Cofiman et al., no single minor complication (superficial wound infection, delayed union etc.) was detected in this treatment Group II. In contrast, in Group I treated with the DePuy Synthes^®^ one-third semitubular plate with a rather greater incision, four minor complications (22%) occurred. In terms of major complications, such as secondary loss of reduction, non-union, major wound infections, etc., only one major complication was detected in both groups without reaching significance (Group I 5.5%, Group II 4.7%. See Table 3).

Considering the consistently low complication rates in the above mentioned studies (White et al., Cofiman et al. [22,27]) as well as in the presented study for the patients treated with the IM IlluminOss^®^ Photodynamic Bone Stabilization System, we are convinced that the significantly reduced size of the surgical incision wound plays a decisive role in the reduction of wound healing disorders, especially in older patients with significant comorbidities. Moreover, our data suggest that the small surgical incision allows for an overlook of the posttraumatic soft-tissue swelling and is not considered as a contraindication to a potentially earlier surgery, along with earlier mobilization and an earlier discharge from the hospital.

Early weight bearing and postoperative mobilization are very controversially discussed in the literature. In this context, patient-related factors, such as age, comorbidities, and fracture morphology, have a strong impact on the postoperative treatment [14,33,45,48,59].

Smeeing et al. showed in a normal-aged patient population (18–65 y) without comorbidities suffering from Lauge-Hansen supination external rotation type 2–4 ankle fractures treated with ORIF that a postoperative unlimited weight-bearing and mobilization regimen improved short-term functional outcome after six weeks, shortened the absence from work, and improved the time to return to sports compared to the patients who only followed a limited weight-bearing or unprotected non-weight-bearing therapy regimen. The mean OMAS (61.2 ± 19.0) after six weeks was up to 10 to 15 points higher compared to the two other groups [35].

In this context, we were able to prove in the actual study that treatment of distal fibula fractures with IM stabilization using the innovative IlluminOss^®^ Photodynamic Bone Stabilization System allowing for an immediate weight bearing in elder patients with a wide range of comorbidities leads to similar good functional results six weeks postoperatively, as described by Smeeing in patients under 65 (see Table 4). Therefore, the actual data present a significant better outcome at the first two follow-up exams (6 and 12 weeks postoperatively) compared to the ORIF group who followed a restricted postoperative rehabilitation protocol including partial weight bearing (see Table 1).

We believe this new IM implant used in the presented study allows for a safe and stable fracture fixation, and it is the key to the early full weight bearing and good clinical outcome in the enrolled elderly patients with significant comorbidities.

Regarding the hospital length of stay, a significant difference between the two groups was identified in favor of Group II (*p* = 0.05, see Table 6). This result confirmed the significant differences between patients from Group I and Group II in terms of range of motion and the assessed outcome scores (see Table 4). In addition, patients in Group II had a significantly shorter preoperative delay caused by posttraumatic swelling of the ankle compared to patients in Group I (*p* = 0.01, see Table 6).

The outcome scores assessed in the presented study are self-assessment questionnaires that reflect the subjective physical well-being and clinical outcome of the individual patients [60]. All used outcome scores have a graduation divided into five scales, ranging from poor to fair over good to excellent. Especially in the early follow-up exams after 6 and 12 weeks of the postoperative treatment, functional ankle scoring results showed a very significant difference (OMAS *p* = 0.01, KPSS *p* = 0.01 and 0.02 see Table 4) for the used scores in favor of Group II (see Table 4). No statistical differences regarding the used outcome scores were detected in the follow up examination after 6 and 12 months (OMAS *p* = 0.06/0.07, KPSS *p* = 0.06/0.06, see Table 4).

The distinct lack of comparability due to different applied postoperative treatment regimens is considered a basic limitation. While Group I patients (semitubular plate) were treated with partial weight bearing for 6 weeks, Group II patients (IlluminOss^®^ Photodynamic Bone Stabilization System) started full weight bearing right after surgery. However, recommendations for patients treated with the new intramedullary implant are still missing the guidelines of the DGOU, as well as of the AO, for patients treated with semitubular plates involving partial weight bearing [59]. The allowance of full weight bearing led to an improved clinical outcome in patients treated with the intramedullary Photodynamic Bone Stabilization System. The presented results seem to be reliable and safe, and they should be considered an important contribution of our research.

Due to the small number of patients, the influence of implant removal was not analyzed. However, the presented follow-up rate of 86.5%, the wide assessment of functional parameters, and the prospective randomized character of the study certainly present the strengths of the presented work.

Studies with higher sample sizes are necessary in the future to demonstrate the benefits and possible disadvantages of these novel implant systems in the treatment of ankle fractures. Although we were able to show in our clinical prospective study for the first time a comparison of outcomes in geriatric patients for both implant groups, this study has its limitations. The fact that in our population, no fracture-related or implant-related infection occurred in the Photodynamic Bone Stabilization System, thus there was no necessity for implant removal in this group, does not mean that this complication is impossible. The used blue light might have antibacterial effects that must be investigated in future studies. Nevertheless, there is still the question to answer of how to remove this implant. An implant removal kit is provided on the market by the manufacturer, but still only a few cases of implant removal are known. Further investigations of possible complications in the usage of this novel implant have to be done in the future.

Although the current literature shows a trend towards the use of locking plate systems in distal fibula FX, and especially in osteoporotic FX, we showed that the use of this new intramedullary system provides a benefit for the geriatric population regarding postoperative clinical outcome, as well as shorter time period to full recovery and full weight bearing, plus very gentle soft tissue management due to the small incision site as compared to the locking plate treatment [49,61,62]. For this study, the use of locking one-third semitubular plates instead of the applied non-locking one-third semitubular plates could have been beneficial for patients randomized in Group I. Thus far, it could be shown that the use of locking plates allows an early weight bearing in younger patients suffering from a distal fibula fracture [20,35]. Nevertheless, the extended surgical approach and operative soft tissue stress can pose a disadvantage in geriatric patients and lead to soft tissue problems as compared to minimally invasive surgical methods; hence, minimally invasive surgical treatment should experience a renaissance [63,64,65]. Overall, all geriatric patients will benefit individually from a faster recovery, but also the cost of medical care could be reduced in general due to shorter hospital stays. Nevertheless, this study focused on the patient’s outcome; therefore, an additional economic analysis could provide supplementary information regarding cost effectiveness. In addition, an analysis of the assumed reduction of health-related costs is advisable.

In general, adding two more groups to the study—e.g., (a) conservative treatment and (b) current intramedullary fibular nail—would add an interesting additional aspect regarding the treatment of distal fibula fractures in geriatric patients, as well as provide broader coverage of all aspects in the treatment of such fractures.

## 5. Conclusions

The presented results demonstrate that the Photodynamic Bone Stabilization System for treating distal fibula fractures in elderly patients with significant comorbidities leads to good clinical results. In fact, the immediate postoperative weight bearing showed that the FX stabilization with this new intramedullary system is a safe method for treating these fragile FXs. However, in comparing the treatment of distal fibula fractures using semitubular plates with the innovative IM system, the first two follow-up exams after 6 and 12 weeks raised our confidence that this new implant system provides a safe and stable treatment option. In addition, the patients treated with this new implant will benefit from the possibility of immediate postoperative full weight-bearing and, therefore, from a faster return to their normal daily life and freedom.

In summary, the successful treatment of distal fibula fractures in elderly patients is essentially dependent on sufficient wound healing and high primary stability to allow for immediate postoperative full weight bearing and initial functional rehabilitation.

## Figures and Tables

**Table 1:** Rehabilitation protocols for both study groups.

**Rehabilitation Protocol**	**Week 1–6 after Surgery**	**Week 7–12 after Surgery**
**DePuy Synthes^®^** **One-Third Semitubular Plate**	partial weight bearing 20 kg	increase weight bearing load
	Walking boot	train away the walking boot
	crouches	crouches till full weight bearing
	pain-adapted motion without limitation	pain-adapted motion without limitation
	**Week 1–3 after Surgery**	**Week 4–12 after Surgery**
**IlluminOss^®^ Photodynamic Bone Stabilization System**	full weight bearing	full weight bearing
	Walking boot	train away the walking boot and switch to an ankle brace
	no crouches	no crouches
	pain-adapted motion without limitation	pain-adapted motion without limitation

**Table 2:** Patient demographics and injury characteristics of both groups are shown. Data are provided as ∅ mean and +/− standard deviation.

Characteristics	DePuy Synthes^®^ One-Third Semitubular Plate	IlluminOss^®^ Photodynamic Bone Stabilization System
Mean age (years)	∅76 ± 8	∅80 ± 8
Sex (male: female)	8:10	7:14
Side (right:left)	10:8	10:11
Mean BMI	∅26	∅24
Mean CCI	∅2 ± 1	∅2 ± 1
**ASA Scoring**		
ASA I	2	3
ASA II	13	12
ASA III	3	6
Surgery time (minutes)	∅45 ± 5	∅36 ± 4

**Table 3:** Overview of the complications for both patient groups, Group I treated with DePuy Synthes one-third semitubular^®^ plate and Group II treated with IlluminOss^®^ Photodynamic Bone Stabilization System.

Minor Complication	DePuy Synthes^®^ One-Third Semitubular Plate	IlluminOss^®^ Photodynamic Bone Stabilization System	*p*-Values
Swelling/redness	2 (11%)		
deep vein thrombosis	1 (5.5%)		
respiratory infection	1 (5.5%)		
			***p* = 0.01**
**Major Complication**			
deep wound infection	1 (5.5%)		
Loss of reposition		1 (4.7%)	
			*p* = 0.47

**Table 4:** Functional Ankle Scoring was assessed using VAS, OMAS, and KPSS for both treatment/patient groups separately at 6 and 12 weeks as well as 6 and 12 months postoperatively. Results are given as ∅ mean and +/− standard deviation. Group I was treated by DePuy Synthes one third semitubular, Group II with the IlluminOss^®^ Photodynamic Bone Stabilization System.

Treatment Group	Scores	6 Weeks	12 Weeks	6 Months	12 Months
	**VAS**				
Group I		4 ±.1	2.94 ± 0.89	2.41 ± 1.00	1.94 ± 0.65
Group II		3.19 ± 0.9	2.52 ± 0.92	2.2 ± 0.76	1.70 ± 0.73
	*p* value	**0.01**	0.09	0.24	0.15
	**OMAS**				
Group I		∅44.3 ± 18	∅55.9 ± 16	∅63.8 ± 15	∅70.3 ± 8
Group II		∅63.7 ± 12	∅67.5 ± 14	∅71.2 ± 10	∅76.2 ± 10
	*p* value	**0.01**	**0.01**	0.06	0.07
	**KPSS**				
Group I		∅40.61 ± 10	∅52.30 ± 11	∅66.52 ± 12	∅69.98 ± 13
Group II		∅57.04 ± 10	∅61.17 ± 9	∅72.3 ± 11	∅76.63 ± 9
	*p* value	**0.01**	**0.02**	0.06	0.06

**Table 5:** Range of Motion at each single follow-up exam. Results are given as ∅ mean and +/− standard deviation. Group I was treated by DePuy Synthes one third semitubular, Group II with the IlluminOss^®^ Photodynamic Bone Stabilization System.

ROM	6 Weeks	12 Weeks	6 Months	12 Months
**Group I**				
Extension(dorsiflexion)	∅4 ± 2	∅10 ± 3	∅14 ± 2	∅18 ± 2
Flexion	∅20 ± 4	∅23 ± 4	∅32 ± 4	∅35 ± 3
**Group II**				
Extension(dorsiflexion)	∅10 ± 2	∅15 ± 4	∅18 ± 4	∅18 ± 2
Flexion	∅25 ± 4	∅32 ± 3	∅34 ± 4	∅36 ± 2
*p* value				
Extension(dorsiflexion)	**0.01**	**0.01**	0.03	0.47
Flexion	**0.01**	**0.01**	0.07	0.19

**Table 6:** Interval between trauma and surgery as well as mean length of hospital stay for both groups.

**Interval between Trauma and Surgery**	**Days**
general	∅8 days
DePuy Synthes^®^ one-third semitubular plate	∅9 days
IlluminOss^®^ Photodynamic Bone Stabilization System	∅4 days
	***p* = 0.01**
**Length of Hospital Stay**	**Days**
general	∅6 days
DePuy Synthes^®^ one-third semitubular plate	∅9 days
IlluminOss^®^ Photodynamic Bone Stabilization System	∅5 days
	***p* = 0.05**

## Data Availability

Availability of data and materials. To request the raw data: the first author of the manuscript can be contacted: Michael Zyskowski, Department of Trauma Surgery, Klinikum rechts der Isar, Technical University of Munich, Ismaninger Strasse 22, 81675 Munich, Germany, Tel.: 0049-89-4140-2126, e-mail: michael.zyskowski@mri.tum.de.

## Abbreviations

DGOU: German Society for Orthopedics and Trauma, FAOS: Foot and Ankle Outcome Score, Fx: Fracture, IM: intramedullary, KPSS: Karlsson and Peterson Scoring System for Ankle function, OMAS: Olerud-Molander Ankle Score, ORIF: Open reduction internal fixation.
